# The enhancement of enzymatic hydrolysis of lignocellulosic substrates by the addition of accessory enzymes such as xylanase: is it an additive or synergistic effect?

**DOI:** 10.1186/1754-6834-4-36

**Published:** 2011-10-05

**Authors:** Jinguang Hu, Valdeir Arantes, Jack N Saddler

**Affiliations:** 1 Forestry Products Biotechnology/Bioenergy Group, Wood Science Department, University of British Columbia, 2424 Main Mall, Vancouver BC, V6T 1Z4, Canada

## Abstract

**Background:**

We and other workers have shown that accessory enzymes, such as β-glucosidase, xylanase, and cellulase cofactors, such as GH61, can considerably enhance the hydrolysis effectiveness of cellulase cocktails when added to pretreated lignocellulosic substrates. It is generally acknowledged that, among the several factors that hamper our current ability to attain efficient lignocellulosic biomass conversion yields at low enzyme loadings, a major problem lies in our incomplete understanding of the cooperative action of the different enzymes acting on pretreated lignocellulosic substrates.

**Results:**

The reported work assessed the interaction between cellulase and xylanase enzymes and their potential to improve the hydrolysis efficiency of various pretreated lignocellulosic substrates when added at low protein loadings. When xylanases were added to the minimum amount of cellulase enzymes required to achieve 70% cellulose hydrolysis of steam pretreated corn stover (SPCS), or used to partially replace the equivalent cellulase dose, both approaches resulted in enhanced enzymatic hydrolysis. However, the xylanase supplementation approach increased the total protein loading required to achieve significant improvements in hydrolysis (an additive effect), whereas the partial replacement of cellulases with xylanase resulted in similar improvements in hydrolysis without increasing enzyme loading (a synergistic effect). The enhancement resulting from xylanase-aided synergism was higher when enzymes were added simultaneously at the beginning of hydrolysis. This co-hydrolysis of the xylan also influenced the gross fiber characteristics (for example, fiber swelling) resulting in increased accessibility of the cellulose to the cellulase enzymes. These apparent increases in accessibility enhanced the steam pretreated corn stover digestibility, resulting in three times faster cellulose and xylan hydrolysis, a seven-fold decrease in cellulase loading and a significant increase in the hydrolysis performance of the optimized enzyme mixture. When a similar xylanase-aided enhancement strategy was assessed on other pretreated lignocellulosic substrates, equivalent increases in hydrolysis efficiency were also observed.

**Conclusions:**

It was apparent that the 'blocking effect' of xylan was one of the major mechanisms that limited the accessibility of the cellulase enzymes to the cellulose. However, the synergistic interaction of the xylanase and cellulase enzymes was also shown to significantly improve cellulose accessibility through increasing fiber swelling and fiber porosity and also plays a major role in enhancing enzyme accessibility.

## Background

A crucial step in the bioconversion of lignocellulosic feedstocks to biofuels is to cost-effectively maximize the saccharification of the cellulose and hemicellulose components to fermentable sugars. One of the challenges is the still too high enzyme costs involved in the saccharification of the cellulosic component [1,2] and, to a lesser extent, the loss of some of the hemicellulosic sugars during pretreatment [3]. Thus, in many pretreatment strategies such as steam explosion, mild severity conditions are often used to avoid, or at least minimize, sugar loss during pretreatment [4]. Under these milder pretreatment conditions, some of the hemicellulose, mostly xylan in agricultural residues and hardwood, remains associated with the cellulosic-rich water insoluble fraction [5]. However, this residual hemicellulose component is known to exert a significant influence on the effectiveness of enzymatic hydrolysis of its cellulosic component [6-10].

The hemicellulose-degrading enzyme activities detected in most of the commercially available cellulase preparations are too low, or are insufficiently active enough, to achieve significant conversion of the residual hemicellulose [11,12]. Therefore, supplementation of the cellulase enzymes with the so called 'accessory enzymes', such as xylanase-enriched preparations, has been the most common approach to increase the overall fermentable sugar yields from pretreated hardwoods [8,13] and agricultural residues [12,14]. Although accessory enzymes offer the potential to increase substrate digestibility, the high dosages of enzyme supplementation applied in many of the past studies can be difficult to justify because of the increased enzyme costs that are incurred. It is generally acknowledged that among the several factors that hamper our current ability to attain efficient lignocellulosic biomass conversion yields at low enzyme loadings, a major problem lies in our incomplete understanding of the cooperative action of the different enzymes acting on pretreated lignocellulosic substrates.

Hemicellulose, which is generally found in higher concentrations on the outer surface of cellulose fibers but is also diffused into the interfibrillar space through fiber pores [15,16], has been proposed to act as a physical barrier that limits the accessibility of the cellulase enzymes to the cellulose [7,8,17,18]. In recent work, we and other workers have also shown that the limited access of cellulase enzymes to the cellulose chains is a key factor which necessitates the use of relatively high enzyme dosages to attain effective cellulose hydrolysis [19]. One of the main beneficial effects of cellulase supplementation with xylanase during biomass saccharification is thought to be the result of the improved cellulose accessibility as a result of xylan solubilization [6,12].

It has been suggested that xylan forms a sheath on each cellulose microfibril and that it is also 'zipped into' the cellulose microfibrils during crystallization, shortly after cellulose synthesis [20,21]. Thus it might be anticipated that the depolymerization of cellulose by cellulases within the fiber would expose the xylan chains naturally trapped within or between microfibrils to the action of xylanase. If true, this degradation model might indicate the potential synergism between xylanase and cellulase enzymes during lignocellulose hydrolysis. Previous work looking at xylanase-aided bleaching treatment of Kraft cellulosic pulps showed that the xylanase not only hydrolyzed the reprecipitated xylan on the fiber surface but also increase pulp fiber porosity, resulting in a substantial increase in the permeability of the cellulosic pulp fibers [18,22,23].

The synergistic action among the multiple forms of hemicellulose-degrading enzymes (for examples, enzymes acting on the xylan backbone and on xylan side chains) and also among the cellulose-degrading enzymes (such as exoglucanases and endoglucanases) has been studied extensively [9,24-26]. Although synergistic cooperation between cellulases and an endoxylanase have been observed at low substrate conversion yields [9,27,28], limited work has looked at the interaction between xylanase and cellulases at conditions relevant to the biofuels industry. This is when relatively low enzyme loadings are used to achieve fast and nearly complete hydrolysis of the cellulose. Similarly, the lack of relevant controls in this previous body of work has made it difficult to determine if the beneficial effect of the accessory enzyme addition was a result of a cooperative interaction (synergism) with the cellulases or merely an additive effect, as the increased substrate hydrolyzability upon enzyme supplementation was typically associated with a corresponding and often substantial increase in protein loading.

In the work reported here, the possible additive or synergistic interaction of xylanase with cellulases was determined during the hydrolysis of steam pretreated corn stover (SPCS) at the minimum enzyme loading required to achieve substantial (greater than 70%) cellulose hydrolysis. Initially, two strategies were evaluated; xylanase supplementation of the minimum cellulase dose required for effective cellulose hydrolysis, and replacement of a portion of the minimum cellulase dose with xylanase while keeping the total protein loading constant. The degree of synergism (DS) was calculated and compared at various xylanase:cellulase ratios. To further investigate the xylanase-cellulase interaction mechanisms, hydrolysis experiments were also carried out by adding the enzymes (cellulases and xylanase) separately, simultaneously and sequentially. The changes in the gross fiber characteristics of SPCS were also monitored over the course of hydrolysis with or without xylanase addition. Finally, the beneficial hydrolysis-boosting effect of xylanase was further evaluated on a range of lignocellulosic substrates. In all of the hydrolysis experiments, controls containing BSA and additional cellulolytic enzymes at equivalent protein loadings to the xylanase were carried out in an attempt to evaluate whether xylanolytic activity offers any advantage over simply increasing the protein dose or the overall cellulase activity.

## Results

### The protein content and specific activities of the enzyme preparations

Initially, the protein concentrations and specific activities of the three commercial glycoside hydrolase preparations used in this study were determined and compared (Table 1). As expected, all preparations demonstrated substantial differences in their protein content and specific activities towards model substrates. Novozyme 188, which is commonly used as β-glucosidase supplementation, displayed the highest protein concentration and β-glucosidase activity. The cellulase enzymes cocktail Celluclast 1.5 L showed the highest activity towards all of the cellulosic substrates, while the other enzyme preparations had below detectable levels of filter paper activity and contained very low carboxymethyl cellulase (CMCase) activity. The Celluclast preparation contained low levels of endoxylanase activity, indicating its low hydrolytic capability towards xylan. In contrast, the Multifect Xylanase showed very high endoxylanase activity relative to the other enzyme preparations and very low activities towards the cellulosic substrates, indicating its overall low saccharification activity towards cellulose.

**Table 1:** Protein content, filter paper activity and specific activities of the commercial enzyme preparations on model substrates

Enzyme preparation	Protein content (mg/mL)	FPA (FPU/mL)	CMCase (U/mL)	β-glucosidase (U/mL)	CBH 1 (U/mL)	Xylanase (U/mL)	β-xylosidase (U/mL)
Celluclast 1.5L	129.2	50.3	474.7	17.0	158.0	438.8	37.8
Multifect Xylanase	37.1	n/a	9.0	12.7	2.7	2588.4	22.5
Novozym 188	233.4	n/a	15.0	239.0	26.2	32.63	3.9

CMCase: carboxymethyl cellulase; CBH1: cellobiohydrolase 1; FPA: filter paper activity; FPU: filter paper unit; n/a: negligible activity detected.

### Effect of xylanase supplementation on the enzymatic hydrolysis of SPCS

**Figure 1:**
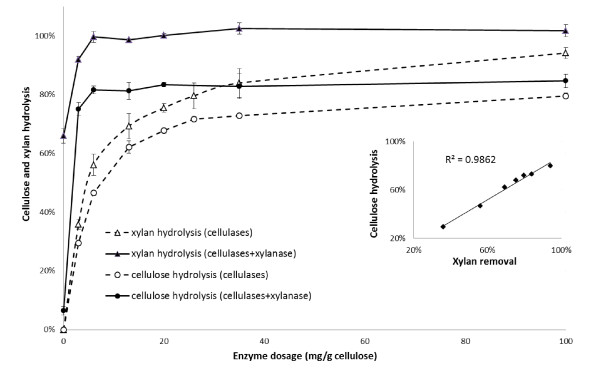
**Conversion of SPCS at increasing cellulase doses with or without xylanase supplementation (60 mg/g cellulose) after 72 h hydrolysis**. Relationship between xylan removal and cellulose conversion after 72 h hydrolysis at various enzyme doses (inset). Full line: hydrolysis in the presence of xylanase; dashed line: hydrolysis in the absence of xylanase. SPCS: steam pretreated corn stover.

In order to assess the influence of xylanase addition during the enzymatic hydrolysis of the SPCS substrate, the hydrolysis experiments were carried out over a range of increasing dosage of cellulase activity (Celluclast) in the absence and presence of fixed amounts of xylanase activity (Multifect Xylanase) (Figure 1).

In the absence of xylanase, cellulose and xylan hydrolysis increased with increasing cellulase loading and reached a plateau at a cellulase protein loading of 35 mg/g cellulose (Figure 1). At this enzyme dosage, the cellulose and xylan hydrolysis were about 70% and 80%, respectively. A further increase in cellulase loading beyond 35 mg/g cellulose resulted in only marginally improved hydrolysis yields. Alternatively, when the SPCS substrate was hydrolyzed with increasing cellulase loading in the presence of xylanase (60 mg/g cellulose), a significant increase in cellulose hydrolysis was observed, with more than 80% of the cellulose hydrolyzed at a cellulase loading of 5 mg/g cellulose, compared to only about 45% in the absence of xylanase supplementation. As expected, xylan hydrolysis also increased significantly from 56% in the absence of xylanase supplementation to over 95% with xylanase supplementation. Further increasing the cellulase loading beyond 5 mg/g cellulose in the presence of xylanase supplementation did not significantly increase either cellulose or xylan hydrolysis.

Interestingly, the addition of xylanase (60 mg/g cellulose) alone, in the absence of cellulases, resulted in only 60% of the xylan being hydrolyzed and further increases in xylanase loading did not improve the hydrolysis of the xylan (data not shown). However, the addition of low amounts of cellulases (5 mg/g cellulose) significantly increased xylan hydrolysis (Figure 1).

It appears that the supplementation of cellulases with xylanase not only substantially increased the rate of hydrolysis of both the cellulose and xylan, it also increased the extent of hydrolysis. For example, the maximum degree of cellulose and xylan hydrolysis in the absence of xylanase supplementation were about 75% and 93%, respectively, at a cellulase loading of 95 mg/g cellulose, whereas cellulose and xylan hydrolysis were about 80% and 100%, respectively, when xylanase was added (60 mg/g cellulose), even at relatively low cellulase loadings of 5 mg/g cellulose. This increase suggested that the residual xylan present in the pretreated SPCS substrate played an important role in limiting the ease of cellulose hydrolysis.

### Cellulase replacement with xylanase versus cellulase supplementation with xylanase

Although it was clear that xylanase supplementation improved both cellulose and xylan hydrolysis at various levels of cellulase loading (Figure 1), it appeared that a significant xylanase-boosting effect was only achieved when low cellulase loadings were used. To further evaluate the interaction between the cellulase and xylanase enzymes during hydrolysis of SPCS, two sets of hydrolysis were carried out. In the xylanase supplementation approach, varying amounts of xylanase (5 to 60 mg/g cellulose) were added to the minimum amount of cellulases (35 mg/g cellulose) which had previously been determined to be required for 70% of the cellulose to be hydrolyzed (Figure 1). In the cellulase replacement approach, varying amounts of the cellulases were replaced with xylanase (up to 86% cellulase replacement on a protein basis) while the total amount of enzyme added, on a protein basis, was kept constant at 35 mg/g cellulose.

It was apparent that supplementing the cellulases with varying amounts of xylanase increased both the cellulose and xylan hydrolysis (Table 2). However, the DS between cellulases and xylanase was about 1, indicating that the observed improvement in SPCS hydrolyzability was more a product of increased enzyme loading. This could be termed an additive effect, as the saccharification performance of the unsupplemented cellulase mixture was 22.8 mg sugars/mg enzymes while the saccharification performance of the xylanase-supplemented cellulase mixture was 21.5 mg sugars/mg enzymes. Alternatively, when relatively small amounts of the cellulases were replaced with xylanase, a slight increase in the degree of synergism was observed, suggesting synergistic cooperation between the cellulase and xylanase enzymes (Table 2). Although lower amounts of cellulase enzymes were present under these conditions (71% cellulase and 29% xylanase) as compared to hydrolysis runs with no cellulase replacement (100% cellulase, 35 mg/g), the same levels of SPCS hydrolysis were obtained (74% cellulose and 82% xylan hydrolysis, Table 2). As the percentage of cellulases replaced by xylanase was increased (up to 86%), the degree of synergism also increased. Under the conditions tested, the highest degree of synergism was observed at a cellulase and xylanase loading of 5 and 30 mg/g cellulose, respectively, which resulted in a substantial increase in both cellulose and xylan hydrolysis (86% cellulose and 99% xylan hydrolysis, Table 2). It appears that with this enzyme ratio, the xylanase and cellulases worked synergistically to hydrolyze the SPCS, resulting in an enzyme mixture with a relatively high saccharification performance (27.3 mg sugars/mg enzymes). Similar SPCS hydrolysis yields could be achieved by replacing cellulases with xylanase to a total final protein loading of 35 mg/g cellulose, as compared to increasing the enzyme dosage by supplementing the 35 mg cellulases/g cellulose with increasing amounts of xylanase (Table 2). As the enzyme mixture containing 5 mg of cellulases/g cellulose and 30 mg xylanase/g cellulose displayed the highest degree of synergism (1.62) during the hydrolysis of the SPCS substrate, this mixture was used to try to better understand the mechanism behind this observed synergism.

**Table 2:** Effect of cellulase supplementation with xylanase and cellulase replacement with xylanase on cellulose and xylan hydrolysis, and on the degree of synergism during hydrolysis of SPCS after 72 h

Hydrolysis strategy	Total protein (mg/g cellulose)	Enzyme mixture	Xylanase (% total protein preparation)	DS	Cellulose hydrolysis (%)	Xylan hydrolysis (%)
Xylanase supplementation	40	35 mg C + 5 mg X	13	0.99	73.9	80.1
	45	35 mg C + 10 mg X	22	0.96	74.1	83.6
	45	35 mg C + 10 mg BSA	0	n/a	72.9	81.3
	95	35 mg C + 60 mg X	63	1.02	87.1	100

Cellulase replacement	35	35 mg C	0	n/a	72.1	81.6
		25 mg C + 10 mg X	29	0.98	74.1	82.6
		15 mg C + 20 mg X	57	1.09	74.2	82.6
		10 mg C + 25 mg X	71	1.31	82.3	98.6
		5 mg C + 30 mg X	86	1.62	86.3	99.3
		5 mg C + 30 mg BSA	0	n/a	55.8	68.6

Separate hydrolysis	5	5 mg C	0	n/a	45.6	56.3
	30	30 mg X	100	n/a	5.2	61.4

Separate hydrolysis used as control. BSA: bovine serum albumin; C: cellulases; DS: degree of synergism; SPCS: steam pretreated corn stover; X: xylanase; n/a: not applicable

### Simultaneous, separate, and sequential hydrolysis

**Figure 2:**
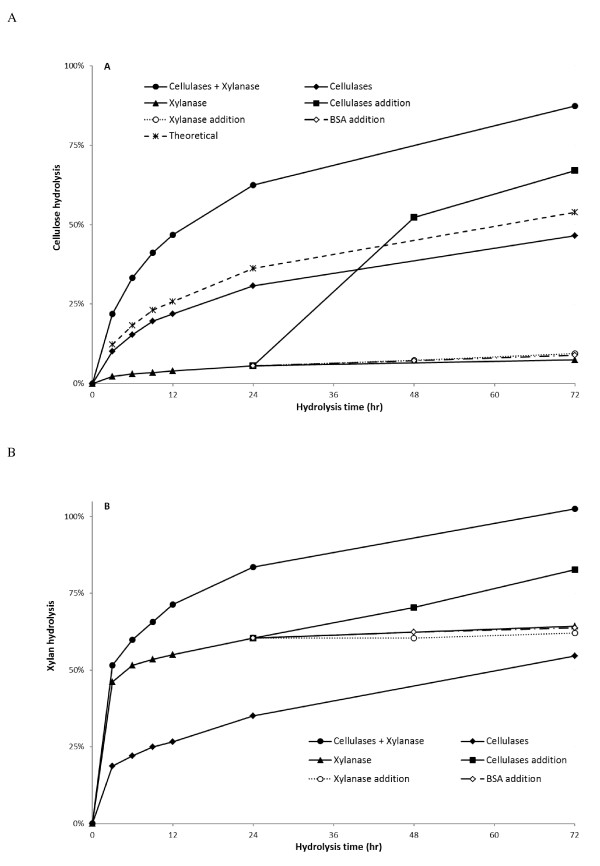
**Time course of SPCS hydrolysis**. Separate hydrolysis: (black rhombus) 5 mg cellulases or (black triangle) 30 mg xylanase; Simultaneous hydrolysis: (black circle) 5 mg cellulases and 30 mg xylanase; Sequential hydrolysis: addition of (black square) 5 mg cellulases, (clear circle) 5 mg xylanase and (clear rhombus) 5 mg BSA to pre-hydrolyzed SPCS with 30 mg xylanase for 24 h. Theoretical: (asterisk) sum of cellulose conversion after hydrolysis with 30 mg xylanase and 5 mg cellulases separately. **(A) **Cellulose hydrolysis. **(B) **Xylan hydrolysis. BSA: bovine serum albumin; SPCS: steam pretreated corn stover.

Most of the previous work in this area has reported an increase in substrate digestibility upon xylanase supplementation, without further explanation of the likely mechanism behind the cooperative interaction between both types of enzymes. To try to further clarify the mechanisms behind the observed synergistic cooperation between the cellulases and xylanase, various time course hydrolysis profiles were followed using simultaneous, separate, and sequential addition of cellulases and xylanase (Figure 2).

It was apparent that, regardless of the enzymes addition strategy used, much of the xylan was solubilized during the first three hours of hydrolysis (Figure 2B). The simultaneous (cellulases plus xylanase) and the separate addition (cellulases) strategies showed similar trends of increased xylan removal after 72 hours hydrolysis, resulting in about 100% and 50% xylan solubilization respectively. However, when xylanase was added alone (30 mg/g cellulose), xylan hydrolysis leveled off after 24 hours with only about 60% of the original xylan solubilized. It is likely that the readily accessible xylan was hydrolyzed in the first few hours of hydrolysis and that in order to access the xylan that is more closely associated with the cellulose and 'buried' within the fiber structure, the synergistic interaction with cellulases is required for more extensive xylan solubilization (Figure 2B). The addition of xylanase alone left one third of the original xylan in the SPCS, even if very high xylanase loading (100 mg/g cellulose) was used (data not shown). However, when a sequential cellulase addition approach was used (Figure 2B), the hydrolysis profile followed the same trend as when the cellulases and xylanase were added simultaneously at the beginning of the hydrolysis. However the final xylan hydrolysis yields were lower (Figure 2B).

**Figure 3:**
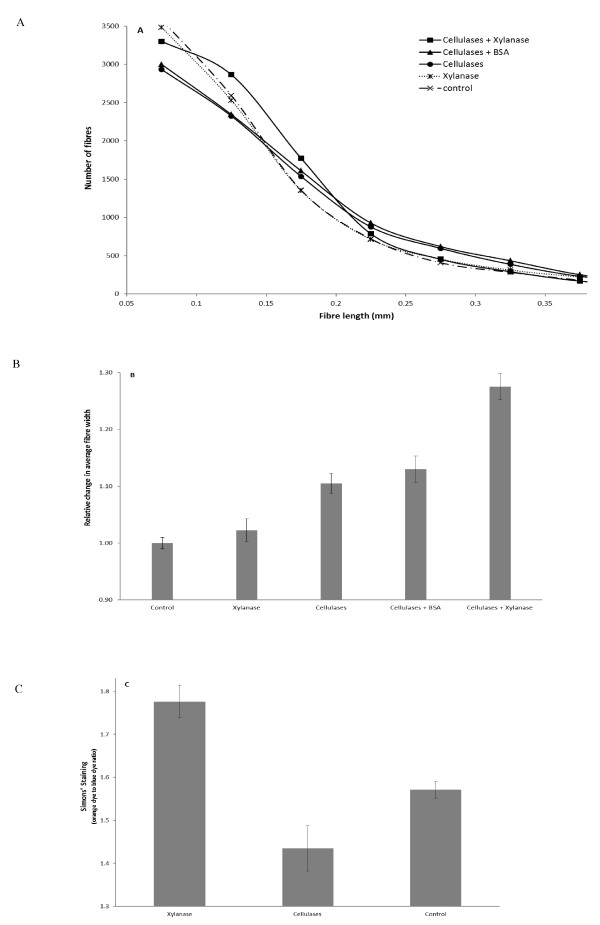
**Change in SPCS fiber properties during separate and simultaneous hydrolysis with cellulases and xylanase after 24 h**. **(A) **Fiber length distribution. **(B) **Average fiber width. **(C) **Fiber surface area (combination interior/exterior) determined by Simons' staining technique. Enzyme loading (mg/g cellulose): cellulases (5), xylanase (30) and BSA (30). Control: substrates were incubated at the same condition without the addition of enzymes. BSA: bovine serum albumin; SPCS: steam pretreated corn stover.

It was apparent that the addition of xylanase alone (30 mg/g cellulose) resulted in limited (5%) cellulose hydrolysis (Figure 3A). However, when cellulases (5 mg/g cellulose) were added to the xylanase pre-hydrolyzed SPCS (sequential hydrolysis, Figure 2A), cellulose hydrolysis increased sharply from 5% at 24 hours of hydrolysis to about 53% at 48 hours, which was significantly higher than the cellulose hydrolysis achieved when adding only cellulases (36% at 48 hours of hydrolysis). This cellulose hydrolysis yield was also higher than would have been the sum of cellulose hydrolysis obtained with cellulases and xylanase when used separately (theoretical conversion). It was apparent that pre-hydrolyzing SPCS with xylanase resulted in enhanced cellulose hydrolysis when cellulases were subsequently added.

When both types of enzymes were added simultaneously (simultaneous hydrolysis), the enzymatic digestibility of SPCS was substantially increased (Figure 2A). For example, when the enzymes were added together, after 12 hours hydrolysis about 50% of the cellulose was hydrolyzed, whereas in the absence of xylanase, only 20% of the cellulose was hydrolyzed. After 72 hours, approximately 45% of the cellulose was hydrolyzed when using cellulase enzymes (5 mg/g cellulose) alone, whereas similar hydrolysis yields could be achieved in just 10 hours when xylanase was supplied together with cellulases at the beginning of hydrolysis. It was apparent that the combined addition of cellulases and xylanase not only resulted in higher hydrolysis rates but also substantially increased the extent of SPCS hydrolysis. After 72 hours, cellulose hydrolysis was almost two-fold higher (87%) as compared to hydrolysis carried out in the absence of xylanase (45%). To ensure that there were no other mechanisms at play, such as a possible 'detergent' effect of added protein, a protein control of BSA was shown to have almost no effect (Figure 2).

### Pulp fiber properties

Hemicellulose is known to contribute to fiber strength and its solubilization is known to influence pulp fiber properties. One method that has been successfully used by the pulp and paper sector to evaluate changes at the fiber level is the use of a fiber quality analyzer (FQA). By using this equipment we hoped to determine any changes in the fiber dimensions and fiber size distribution of the unhydrolyzed and residual SPCS after simultaneous and separate hydrolysis with cellulase and xylanase addition over 24 hours (Figure 3A and 3B).

When compared to the unhydrolyzed SPCS control, xylanase addition alone did not result in any changes in the fiber length distribution (Figure 3A). In contrast, cellulase treatment resulted in a significant decrease in the population of fibers with lengths less than 0.15 mm and also a slight increase in the population of fibers with lengths within the range of 0.15 to 0.35 mm (Figure 3A). The latter group of fibers is likely due to the fragmentation of long fibers into shorter fibers, as the average fiber length after 24 hours decreased from 0.555 mm to about 0.270 mm. Earlier work has shown that the smaller fibers were rapidly hydrolyzed and solubilized [29]. When the xylanase and cellulases were added simultaneously, a different pattern was observed, with a significant increase in the population of fibers in the length range of 0.09 to 0.23 mm (Figure 3A). There was also significantly more fiber fragmentation as the average fiber length was reduced from 0.55 mm to about 0.22 mm. The large amount of fibers in the length range of 0.09 to 0.23 mm was also likely due to the higher fragmentation of SPCS fibers, resulting in the higher numbers of shorter fibers.

We next measured changes in the fiber width of the residual SPCS after hydrolysis, as this value can provide a general indication of the degree of fiber swelling (Figure 3B). As was observed with the fiber length values, xylanase treatment alone promoted only a slight change in the average fiber width, whereas cellulase addition increased the fiber width by about 10%. A significant change in fiber width was observed when both the xylanase and cellulases were added simultaneously, with the fiber width increasing by about 30% as compared to the untreated fibers, and by 20% as compared to the cellulase alone-treated SPCS fibers. This increase in fiber width suggested a significant increase in fiber swelling as a result of the synergistic cooperation of the cellulases and xylanase.

To see if we could quantify any increases in accessibility of the SPCS fibers to the enzymes, we used the Simons' stain method, which has previously been shown to provide a good estimation of cellulose accessibility [19]. An increase in the orange dye (DO) to blue dye (DB) ratio after xylanase treatment indicated that more of the cellulose was accessible, probably due to the removal of the xylan in the SPCS fibers (Figure 3C). The addition of cellulases alone resulted in a decrease in the DO:DB ratio primarily because of the decreasing amount of substrate that was available to measure as hydrolysis proceeded.

### The effect of xylanase supplementation on the enzymatic hydrolysis of other pretreated lignocellulosic substrates

**Figure 4:**
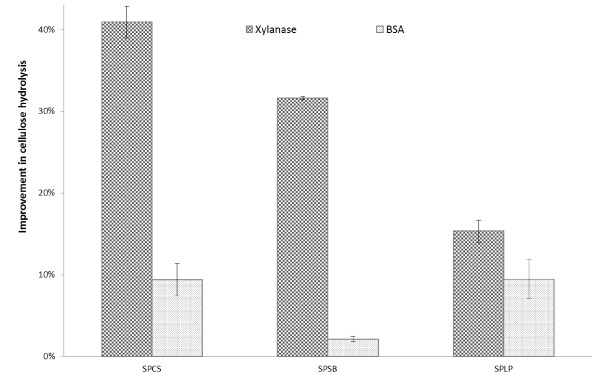
**Improvement in cellulose hydrolysis yields in the presence of xylanase (30 mg/g cellulose) and cellulases (5 mg/g cellulose) as compared to hydrolysis yields in the presence of only cellulases (5 mg/g cellulose) after 72 h hydrolysis of various substrates**: SPCS: steam pretreated corn stover; SPSB: steam pretreated sweet sorghum bagasse; SPLP: steam pretreated lodgepole pine.

To determine if the xylanase-boosting effect observed during hydrolysis of SPCS could also be observed with other biomass substrates, steam pretreated sweet sorghum bagasse (SPSB) and steam pretreated lodgepole pine (SPLP) were hydrolyzed with similar combinations of cellulases and xylanase as were carried out with the SPCS substrate (Figure 4). The SPSB had a higher xylan content than did the SPCS substrate, whereas the xylan content of the SPLP was negligible (Table 3). The effectiveness of hydrolysis of both the SPSB and SPLP substrates was assessed using a total protein loading of 35 mg/g cellulose (5 mg cellulases plus 30 mg xylanase/g cellulose; 5 mg cellulases plus 30 mg BSA/g cellulose). The simultaneous addition of xylanase and cellulases also significantly enhanced the cellulose hydrolysis of both the SPSB and SPLP substrates. Interestingly, xylanase supplementation boosted the cellulose hydrolysis of the SPLP substrate even though the xylan in this substrate was below detectable levels. This suggested that the mechanism behind the xylanase-boosting effect of cellulose hydrolysis in pretreated biomass is not solely due to increasing cellulose accessibility through xylan removal and that it also involves contributions to changes in fiber morphology as was shown with the FQA and Simons' stain values obtained during the hydrolysis of the SPCS substrate.

**Table 3:** Steam pretreatment conditions and chemical composition of pretreated lignocellulosic substrates

Substrate	Pretreatment conditions	Composition of pretreated feedstocks						Abbreviation
			
		*Ara*	*Gal*	*Glu*	*Xyl*	*Man*	*AIL*	
Corn stover	190°C, 5 minutes, 3% SO_2_	1.0	0.7	56.1	7.0	1.1	27.0	SPCS
Sweet sorghum bagasse	190°C, 5 minutes, 3% SO_2_	0.6	0.8	54.3	9.8	1.0	25.8	SPSB
Lodgepole pine	200°C, 5 minutes, 4% SO_2_	bdl	bdl	46.4	bdl	bdl	45.0	SPLP

*AIL*: acid insoluble lignin; *Ara*: arabinan; bdl: below detectable level; *Gal*: Galactan; *Glu*: glucan; *Man*: mannan; *Xyl*: Xylan

## Discussion

Effective enzymatic hydrolysis of the cellulose and hemicellulose present in pretreated lignocellulosic substrates to fermentable sugars has been shown by many workers to require a combination of various glycoside hydrolases whose combined action is believed to be more efficient than the sum of the actions of the individual enzymes. This is the basis of the so called 'synergistic enzyme interaction' effect. Many studies have looked at the synergistic interactions among the major cellulase components and different explanations have been given for their interactions on model cellulosic substrate. Hypotheses include the creation of new hydrolysis sites, removal of physical obstacles [25], presence of stereospecific enzyme activities, and the formation of enzyme-enzyme complexes [24,30,31]. More recently, we and other workers have shown that some accessory enzymes, such as β-glucosidase, xylanase and the cellulase enhancing factors such as GH61, can considerably enhance the hydrolysis effectiveness of cellulase cocktails when added to pretreated lignocellulosic substrates [32-34].

The major beneficial effect of xylanase supplementation to cellulase enzymes mixtures has frequently been suggested to be a result of increasing cellulose accessibility to the cellulase enzymes due to the removal of the xylan coating on the outer surface of the pretreated pulp fibers [6,12]. However, despite the potential to enhance hydrolysis yields by xylanase supplementation, one major reservation is the likely substantial increase in total protein loading that would be required, thus increasing enzyme costs.

In the work described here, our initial approach was to define the minimum amount of cellulase enzymes required for effective hydrolysis and to assess the type and extent of the interaction between xylanase and cellulase enzymes in an attempted to improve hydrolysis efficiency of pretreated lignocellulose at low protein loadings. It was apparent that the type of interaction between xylanase and cellulase enzymes is dependent on the total enzyme loading and enzymes ratio, as has been observed previously [26,35]. A strong synergistic effect was observed at low cellulase loading and when a high xylanase to cellulase ratio was used. The highest DS (1.62) was observed at a xylanase to cellulase protein loading of 6:1, which resulted in a considerable reduction (seven-fold) in the total amount of cellulase enzymes required for effective cellulose hydrolysis of the SPCS substrate. In related work, Bura *et al. *[8] and Berlin *et al. *[11] also found that a similar xylanase to cellulase ratio improved cellulose hydrolysis. However, in these earlier works it was more of an additive effect rather than a truly synergistic interaction.

There are several possible explanations for the strong synergistic interaction observed between cellulases and xylanase. As mentioned earlier, xylanase have been suggested to remove the xylan coat on the surface of pulp fiber, improving cellulose accessibility to cellulase enzymes [6,8,12]. In a related work, Pauly *et al. *[20] found that one third of the xyloglucan present in a dicot plant was entrapped within the microfibril, and its removal required the action of both cellulase and xyloglucanase enzymes. Xylanases have also been suggested to increase the proportion of substrate available for productive cellulase interaction due to their unproductive binding to sites on lignin [36,37]. The xylanase-enriched preparation used in this work belongs to the glycoside hydrolase family 11 (GH11), which lacks a carbohydrate binding module [38], and this enzyme component has been shown to preferentially bind to lignin [39]. Previous work has shown that lignin has very little influence on the xylanase activity as compared to cellulases and β-glucosidase [40]. Therefore, for the work reported here, an increase in cellulase availability due to xylanase binding to lignin is unlikely to be the major reason for the observed enhancement of cellulase activities in the presence of xylanase.

Xylanases have also been shown to result in the solubilization of lignin fractions from pretreated lignocellulosic biomass by breaking down the lignin-carbohydrate complex and consequently improving substrate digestibility [18,22]. It is also worth noting that the xylanase-aided bleaching treatment of cellulosic pulps promoted physical changes to the pulp fibers, such as an increase in fiber porosity and fiber disintegration [18,22,23]. Similar changes during hydrolysis of lignocellulosic biomass are likely to increase the available specific surface area of the cellulose to cellulase enzymes and therefore the effectiveness of the cellulases, a process termed amorphogenesis [41].

Since it has been predicted that a diverse range of plant biomass will be needed to satisfy the projected demands for advanced biofuels [42], the hydrolysis boosting ability of xylanase was also evaluated on a range of cellulosic materials. It was apparent that xylanase treatment could significantly improve the cellulose hydrolysis of all of the lignocellulosic substrates assessed, even for the steam pretreated softwood that contained virtually no xylan. This further supports the proposal that one of the main beneficial effects of the synergistic interaction between xylanase and cellulases is the substantial change in the gross fiber characteristics (for example, fiber swelling) which were observed. It is likely that this synergism occurs in a similar fashion to the amorphogenesis effect that has been suggested in cofactors such as GH61 or cellulose binding modules to enhance the effectiveness of cellulase enzymes [19].

In summary, it appears that the observed xylanase-boosting effect during hydrolysis of pretreated lignocellulosic biomass is a result of both increased cellulose accessibility to cellulase enzymes as a result of xylan removal from pulp fibers, and the synergistic interaction of the xylanase and cellulase enzymes increasing cellulose accessibility through increasing fiber swelling and fiber porosity.

## Conclusion

It was apparent that the overall protein loading required to achieve fast, nearly complete hydrolysis of a model cellulosic substrate (SPCS) could be significantly reduced by making use of the synergistic interaction that occurs between cellulases and xylanase. It is likely that the added xylanase enhanced overall hydrolysis by solubilizing xylan, which impedes access to the cellulose. The 'xylanase-boosting' effect was observed on a range of pretreated lignocellulosic materials, regardless of their xylan content. So called accessory enzymes such as xylanase might offer considerable potential to increase the overall performance of cellulase enzyme mixtures, while reducing the protein loading required to achieve effective hydrolysis of pretreated lignocellulosic substrates.

## Methods

### Lignocellulosic biomass preparation and composition

Corn stover, sweet sorghum bagasse and lodgepole pine were steam pretreated according to previously described procedures [12,43]. The chemical composition of the water insoluble fraction (cellulose-rich material) after pretreatment was determined using the modified Klason lignin method, derived from the TAPPI standard method T222 om-88 [3]. The values determined after the various pretreatment conditions are described in Table 3.

### Enzyme preparations

Cellulases (Celluclast 1.5L, Novozymes, Franklington, NC), from *Trichoderma reesei*, β-glucosidase (Novozym 188, Novozymes A/S, Bagsvaerd, Denamark) from *Aspergillus niger*, and xylanase (Multifect Xylanase, Genencor US Inc., Palo Alto, CA) from a genetically modified strain of *T. reesei *were used. The protein content and enzyme specific activities of the enzyme preparations are summarized in Table 1. The filter paper activity was determined according to International Union of Pure and Applied Chemistry [44]. Xylanase and CMCase activities were determined as described elsewhere [45]. Cellobiohydrolase 1, β-xylosidase and β-glucosidase activities were determined using *p*-nitrophenyl-β-D-cellobioside, *p*-nitrophenyl-β-D-xylopyranoside, and *p*-nitrophenyl-β-D-glucopyranoside as substrates, respectively, as described previously [46]. Protein concentration was measured using the Ninhydrin assay using BSA as the protein standard [47].

### Enzymatic hydrolysis

The hydrolysis experiments were carried out at 2% (w/v) solids loading in sodium acetate buffer (50 mM, pH 5.0). The reaction mixtures (1 mL) were mechanically shaken in an orbital shaker incubator (Combi-D24 hybridization incubator, FINEPCR^®^, Yang-Chung, Seoul, Korea) at 50°C in accordance with previously described methods [19]. Three sets of hydrolysis were carried out. Initially, the required minimum cellulase loading for efficient cellulose hydrolysis in the absence and presence of xylanase (60 mg/g cellulose) and the effect of xylanase were assessed by hydrolyzing SPCS at increasing cellulase loadings (5 to 100 mg/g cellulose). To further assess the mechanism of the xylanase-boosting effect during SPCS hydrolysis, the hydrolysis of SPCS was carried out using two strategies for enzyme addition. In the first, xylanase supplementation, varying amounts of xylanase (0 to 100 mg/g cellulose) were supplemented to the cellulase mixture (35 mg/g cellulose). In the second, cellulase replacement, varying amounts of the total cellulase loading (35 mg/g cellulose) was replaced (up to 86%) with the exact amount of xylanase at a fixed total protein dosage (35 mg/g cellulose). Finally, the SPCS was hydrolyzed with separate, simultaneous, and sequential additions of cellulases (5 mg/g cellulose) and xylanase (30 mg/g cellulose). Sequential hydrolysis was carried out by incubating the SPCS with xylanase for 24 h. Thereafter, cellulases were added to the pre-hydrolyzed mixture and incubated for 48 h. Separate and simultaneous hydrolyzed were carried out over 72 h.

In all of the hydrolysis assays, β-glucosidase was supplemented at a cellobiase units to filter paper units activity ratio of 1:2 to limit end-product inhibition. The addition of xylanase was based on the total cellulose content, as the overall goal of xylanase addition is to enhance cellulose hydrolysis. Protein controls of BSA were used to assess the effect of the addition of a non-hydrolytic protein when compared to the addition of xylanase.

At the end of hydrolysis, samples were heated at 100°C for 10 min to inactivate the enzymes. Supernatants were collected after centrifugation at 13,000 rpm for 10 min. The concentration of glucose and xylose in the supernatants were measured using HPLC (Dionex DX-3000, Sunnyvale, CA) as described elsewhere [48]. The hydrolysis yields of the pretreated substrates were calculated from the cellulose and xylan content as a percentage of the theoretical cellulose and xylan available in the substrates. All hydrolysis experiments were performed in duplicate and mean values and standard deviations are presented.

The following equation was used to calculate the degree of synergism between cellulase enzymes and xylanase during SPCS hydrolysis:

$$\text{DS}=\frac{\text{G}{\text{C}}_{\text{mixture}}}{\sum \text{G}{\text{C}}_{\text{individual}}}$$

where GC_mixture _is the cellulose hydrolysis achieved with cellulases and xylanase added together, and ∑GC_individual _is the sum of cellulose hydrolysis achieved with the individual enzymes.

### Assessment of fiber gross characteristics

To assess changes in fiber characteristics during hydrolysis, a FQA (LDA02; OpTest Equipment, Inc., Hawkesbury, ON, Canada) was used to monitor fiber length and width according to the procedure described previously [49]. The settings on the FQA were adjusted to measure particles down to 0.07 mm, and the fiber length distribution and average fiber width were measured as described previously [19].

The changes in available surface area were determined using a modified version of the Simons' staining technique previously used to evaluate cellulose accessibility to cellulase enzymes [19,50]. The increase in cellulose accessibility was expressed as an increase in the DO to DB ratio.

## List of abbreviations

BSA: bovine serum albumin; CMCase: carboxymethyl cellulase; DB: blue dye; DO: orange dye; DS: degree of synergism; FQA: fiber quality analyzer; HPLC: high performance liquid chromatography; SPCS: steam pretreated corn stover; SPLP: steam pretreated lodgepole pine; SPSB: steam pretreated sweet sorghum bagasse.

## Competing interests

The authors declare that they have no competing interests.

## Authors' contributions

All authors contributed jointly to all aspects of the work reported in the manuscript. JH carried out much of the laboratory work, contributed to planning, interpretation of results and drafting of the paper. VA contributed to the planning, interpretation and drafting. JS contributed to the planning, interpretation and writing of the manuscript. All authors read and approved the final manuscript.
